# Physiologically Based Pharmacokinetic Models in the Prediction of Oral Drug Exposure Over the Entire Pediatric Age Range—Sotalol as a Model Drug

**DOI:** 10.1208/s12248-013-9555-6

**Published:** 2014-01-08

**Authors:** Feras Khalil, Stephanie Läer

**Affiliations:** 1 Institute of Clinical Pharmacy and Pharmacotherapy, Heinrich-Heine University of Düsseldorf, Universitaetsstrasse1, Building. 26.22. Room 02.21, 40225 Düsseldorf, Germany; 2 Institute of Clinical Pharmacy and Pharmacotherapy, Heinrich-Heine University of Düsseldorf, Universitaetsstrasse1, Building. 26.22. Room 02.24, 40225 Düsseldorf, Germany

**Keywords:** administration (oral), child, computer simulation, pharmacokinetics, sotalol

## Abstract

In recent years, the increased interest in pediatric research has enforced the role of physiologically based pharmacokinetic (PBPK) models in pediatric drug development. However, an existing lack of published examples contributes to some uncertainties about the reliability of their predictions of oral drug exposure. Developing and validating pediatric PBPK models for oral drug application shall enrich our knowledge about their limitations and lead to a better use of the generated data. This study was conducted to investigate how whole-body PBPK models describe the oral pharmacokinetics of sotalol over the entire pediatric age. Two leading software tools for whole-body PBPK modeling: Simcyp® (Simcyp Ltd, Sheffield, UK) and PK-SIM® (Bayer Technology Services GmbH, Leverkusen, Germany), were used. Each PBPK model was first validated in adults before scaling to children. Model input parameters were collected from the literature and clinical data for 80 children were used to compare predicted and observed values. The results obtained by both models were comparable and gave an adequate description of sotalol pharmacokinetics in adults and in almost all pediatric age groups. Only in neonates, the mean ratio_(Obs/Pred)_ for any PK parameter exceeded a twofold error range, 2.56 (95% confidence interval (CI), 2.10–3.49) and 2.15 (95% CI, 1.77–2.99) for area under the plasma concentration-time curve from the first to the last concentration point and maximal concentration (C_max_) using SIMCYP® and 2.37 (95% CI, 1.76–3.25) for time to reach C_max_ using PK-SIM®. The two PBPK models evaluated in this study reflected properly the age-related pharmacokinetic changes and predicted adequately the oral sotalol exposure in children of different ages, except in neonates.

## INTRODUCTION

Physiologically based pharmacokinetic (PBPK) models can deliver valuable information during various stages of drug development and research (1–4). Their ability to incorporate information about maturation, growth, and age dependency of anatomical and physiological processes facilitates their use to extrapolate drug pharmacokinetics from adults to children and to explore age-related changes (5,6). In recent years, the implementation of PBPK models in pediatric drug development has become more attractive (7–11), encouraged by an increased awareness of/and interest in pediatric research, especially after the new regulations on medicinal products for pediatric use in both the USA and the European Union (12,13).

Despite the marked potential of PBPK models, uncertainties still exist in the pediatric community about the accuracy of their predictions especially after oral drug administration in pediatric patients of different ages (11). The lack of sufficient published pediatric PBPK models evaluated adequately for the prediction of oral drug absorption and disposition is one main reason. For the intravenous (IV) application, six pediatric whole-body PBPK models evaluated with a total of 10 different drugs have already been reported (4,14–18). By contrast, there is only, to date of writing, two publications with reported pediatric PBPK model predictions evaluated for 6 drugs after oral drug application (4,19), with only one of them evaluated with neonatal experimental data for the modeled drug (19). In addition, there are other reported pediatric PBPK models that were, however, either not evaluated with experimental data (20,21), or focused only on one aspect of the drug pharmacokinetics with no full concentration-time profiles or information about predicted drug absorption or exposure, *e.g*., to predict only the clearance of the drugs (22,23). Given all of these facts, more examples of evaluated pediatric PBPK models for oral drugs are still in demand.

In recognition of this unmet need, a PBPK model drug with an already available large-scale clinical pharmacokinetic data set that covers all pediatric age groups is preferable. Sotalol, an antiarrhythmic drug used in the treatment of supraventricular tachycardia, orally given with >90% bioavailability and almost completely renally eliminated, has been very well studied in both adults and children and fulfills this requirement (24–37). The rich pediatric data of sotalol facilitate a good assessment of the model predictability from adolescents down to neonates, and provide individual full concentration-time profiles with information on drug absorption. Conversely, the rich adult data after both IV and oral administration will enable the validation of the model first in adults before scaling it to children, thus forming a solid basis for age extrapolation. Finally, because commercial PBPK modeling packages are often used nowadays by researchers as a basis for their models, and because no single source for the integrated data does exist, the choice to use two commonly used modeling software tools for model development has been undertaken to minimize bias by software and to examine to what extent the use of different modeling software can influence the obtained results.

This study was made to investigate how whole-body PBPK models, developed using two dedicated PBPK modeling tools, describe the oral pharmacokinetics of sotalol from adults to neonates. A secondary goal was to observe any differences in the performance of these two models across the pediatric age range.

## MATERIALS AND METHODS

### PBPK Modeling Software and Model Parameterization

To develop a whole-body PBPK model of sotalol, two specialized modeling software tools were used separately: software 1 was Simcyp® simulator v.12.1 (Simcyp Ltd, Sheffield, UK) for adults and pediatrics (38), and software 2 was PK-SIM® v.4.2.2 (Bayer Technology Services GmbH, Leverkusen, Germany) with its integrated clearance scaling module (39). In brief, these tools provide a general PBPK model structure to describe drug absorption and disposition in the body and incorporate a large data set of anatomical and physiological parameters with their age dependencies, as permitted by the current scientific knowledge. The detailed structure and methodology of these PBPK models are already published elsewhere (40,41).

However, to complete the model parameterization, the required physicochemical properties of sotalol, along with other drug-dependent parameters, were collected from a comprehensive literature search. Both models used the same values for molecular weight, lipophilicity (octanol–water partition coefficient (logP) value), acid dissociation constant (p*K*_a_), fraction unbound (*f*u), and clearance (CL), as listed in Table I (24,25,31,43,47,60), which summarizes the final model input parameters. The total clearance of sotalol was set to be 0.1125 L h^−1^ kg^−1^ assigned completely as a renal clearance, which is consistent, in its value and the route of elimination, with the literature (24,25,31). The partition coefficients in the tissues were calculated with both software using Rodgers and Rowland’s distribution model (42). The input values assigned to the blood-to-plasma concentration (B/P) ratio differed between software 1 and 2 (1.02 *vs.* 0.86), as these values gave the best visual fit during the IV model development (see Modeling Strategy and Simulation Conditions); however, the models predicted similar *V*_ss_ values, 1.3 and 1.22 L/kg, respectively, in an average adult male individual weighing 70 kg, which are in good agreement with the reported literature values (24,31,43,44). Drug absorption was predicted by the advanced dissolution, absorption, and metabolism (ADAM) model in Simcyp (45), and by a built-in absorption model in PK-SIM (46), with various input measures offered by the two software to account for the drug intestinal permeability. Sotalol is known to be a biopharmaceutics classification system (BCS) Class I drug with high solubility and high permeability profile; however, sotalol *in vitro* measured apparent permeability coefficient (*P*_app_) is very low and does not correlate with the high values of absorbed dose fraction (>90%) obtained from pharmacokinetic studies in humans (47,48). Therefore, the value of the intestinal permeability measure for each model was adjusted separately in order to give the same absorbed fraction and a bioavailability of 90% in adult. Finally, the default mean values used in both models for gastric emptying time (GET) and small intestinal transit time (SITT) were 0.5 and 4 h, respectively.

**Table I:** Input Parameters for Sotalol PBPK Models Using Both Modeling Software

Parameter	Software 1^a^ value	Software 2^b^ value	Reference value	Reference
Molecular weight (g/mol)	272.36	272.36	272.36	PubChem
LogP_(o/w)_	0.37	0.37	0.2, 0.37	PubChem, (47)
Ionization constant	p*K*_a1_ = 8.28	p*K*_a1_ = 8.28	p*K*_a1_ = 8.28	(60)
	p*K*_a2_ = 9.72	p*K*_a2_ = 9.72	p*K*_a2_ = 9.72	
*f*_u_	1	1	1	(24,25,43)
Blood/plasma ratio	1.02	0.86	1.07	(60)
CL_IV, total_	7.875 L/h	0.1125 L h^−1^ kg^−1^	0.09–3.2 L h^−1^ kg^−1^	(31,43,44)
Fraction of renal clearance	100%	100%	90–100%	(24,25)
Permeability measure (cm/s)^c^	2.01 × 10^−4c^	12.6 × 10^−6c^	–	–

*LogP* octanol–water partition coefficient, *fu* fraction unbound, *CL*_*IV*, *total*_ total intravenous clearance, *pK*_*a*_ acid dissociation constant, *pK*_*a1*_ for acidic function, *pK*_*a2*_ for basic function

^*a*^Simcyp®

^*b*^PK-SIM®

^*c*^Human jejunum permeability (*P*_eff_, man) as permeability measure in software 1; *in vitro* intestinal permeability (*P*_app_) as permeability measure in software 2. Both measures were manually adjusted to give the same value of fraction absorbed (*f*_a_) and a bioavailability of 90%

### Pharmacokinetic/Clinical Data

#### In Adults

MEDLINE database was screened for pharmacokinetic studies of sotalol in healthy adults with known age, gender, height or weight, clear dosing information, and available plasma concentration-time profiles. As a result, a total of 27 data sets originating from 11 clinical trials published by 8 different scientific groups (between 1976 and 2010) in 5 countries (26–36) were used in model development and evaluation (Table II) (26–36). Each experimental data set represents a mean observed concentration-time profile in an average of five to six healthy volunteers who received either IV or oral doses of sotalol-HCl. These data were either provided by the author (35,36) or scanned from the publications’ figures (26–34).

**Table II:** Population Characteristics and Dosing Information of the Pharmacokinetic Studies Used in the Development and Validation of the Sotalol Adult PBPK Model

	Applied dose		No. of data sets^a^	Ethnicity	Females (%)	Age range (years)	References
	(mg)	(mg/kg)					
Intravenous sotalol	20	–	1	Caucasian	50	24–53	(26)
	35^b^	0.5	2	Caucasian	50	18–38	(27)
	70^b^	1	1	Asian	0	22–43	(28)
	75	–	1	Caucasian	60	19–45	(29)
	105^b^	1.5	4	Caucasian/Asian	18	18–43	(27,28,30)
	140^b^	2	2	Caucasian	0	21–32	(30,31)
	210^b^	3	2	Caucasian	50	18–38	(27)
Oral sotalol	40	–	1	Asian	0	22–45	(32)
	50	–	1	Asian	0	22–43	(28)
	80	–	2	Caucasian	60	19–45	(29,32)
	100	–	2	Caucasian/Asian	0	22–43	(31)
	160	–	5	Caucasian/Asian	16	22–56	(26,32,33,35,36)
	200	–	1	Asian	0	22–43	(28)
	300	–	1	Asian	0	22–43	(28)
	320	–	1	Caucasian	10	28–56	(34)

^*a*^Each data set represents a mean observed concentration time profile of an average of five to six healthy adult volunteers

^b^Total dose in milligrams was calculated from the reported milligrams per kilogram dose using a reference adult weight value of 70 kg

#### In Children

**Fig. 1:**
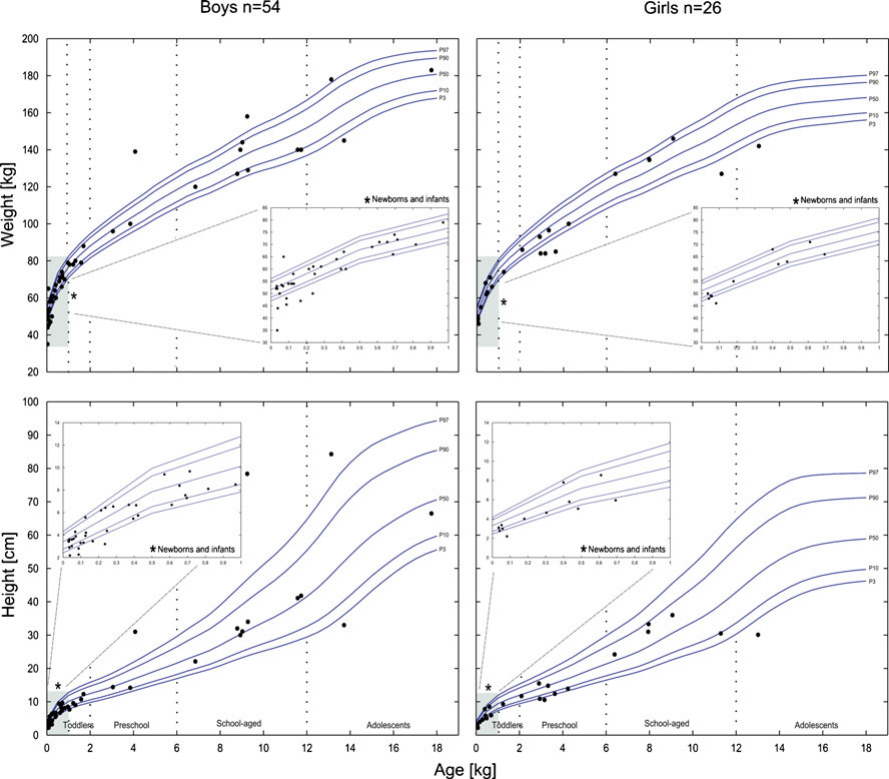
The height and/or weight (*dots*) of each of the 80 children (boys, *n* = 54 (*right*); girls, *n* = 26 (*left*)); the observed population originally exposed to sotalol. In addition, *lines* show pediatric age- and gender-specific percentiles (3rd, 10th, 50th, 90th, and 97th), which represent the normal values of a German representative population (61). *Insets* show the demographics of the segment from birth to the end of the first year to highlight the values of newborns and infants

Eighty pediatric patients of different age groups with known age, gender, height, weight, dosing information, and measured plasma profiles were used. The majority of these data are already published (37). These patients, ranging from age 11 days to 17.7 years (average, 3.51 years, including 13 premature infants) received various doses of sotalol (1.0–9.9 mg kg^−1^ day^−1^) for the treatment of supraventricular tachycardia. The demographics of these children are presented in Fig. 1. This pediatric data set was classified using a system similar to the WHO classification, however, using six different age groups: (a) neonates, 0–28 days (*n* = 14); (b) infants, 1–11 months (*n* = 33); (c) toddlers, 12–23 months (*n* = 6); (d) Preschool-aged, 2–5 years (*n* = 10); (e) school-aged, 6–11 years (*n* = 13); and (f) adolescents, 12–18 years (*n* = 4). This classification was used for presenting results in children.

### Modeling Strategy and Simulation Conditions

**Fig. 2:**
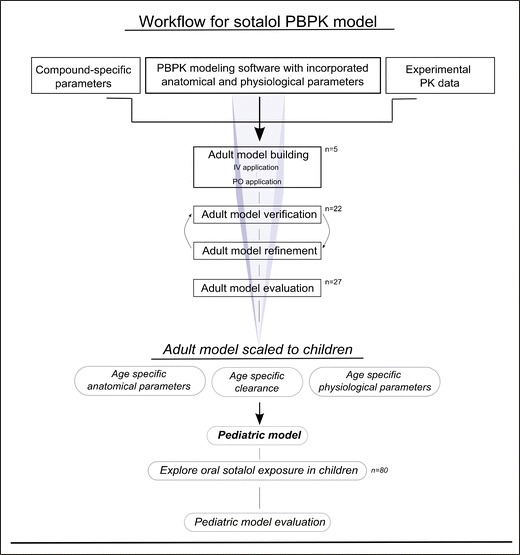
Schematic workflow of the developed PBPK models

The adopted modeling strategy is shown in Fig. 2. An adult model was first developed for the IV application, as this allows for the kinetics of drug disposition to be simulated in the absence of the complexities of the absorption process. Thus, the best set of input parameters, the suitable distribution model, and the most appropriate clearance that collectively gave the best visual description of the observed data used at this stage, were assigned. For the oral application, parameters values from the previous step were kept plus the values of additional parameters that control and influence drug absorption, such as intestinal permeability, GET, and SITT. In the previously mentioned steps (*i.e*., model building), only one fifth (*n* = 5) of the collected adult data set was used, whereas the remaining data (*n* = 22) were used later for a subsequent model verification. The adult model was slightly refined (logP and CL inputs) before the end model evaluation. The final adult model was then scaled down to children, taking into consideration the age dependencies of anatomical and physiological processes/parameters and the ontogeny of clearance pathways, which are already integrated into the modeling software, to predict pediatric sotalol exposure (see also, Clearance Scaling).

The comparison of model results with observed data was based on simulations of virtual populations, where the main results of these simulations are concentration-time profiles. In adults, each virtual population consisted of 100 virtual subjects having the same age range, race, gender composition, and dosing as their respective real population. The resulting mean plasma concentrations were then compared with the mean observed concentrations for model evaluation. Population simulations performed with a higher number of virtual subjects (*n* = 1,000) did not produce any significant difference from the previous ones (using 100 replicates), and did not influence any differences seen between the results of both models. In children, a similar approach was used by performing a population simulation of 100 virtual children each with the same age, race, gender, and dosing information of a real child; however, the resulting median plasma concentrations were used along with the individual observed concentrations in the model evaluation. For all previous simulations, variability ranges for CL, GET, and SITT were assigned to account for the interindividual variability. These values were either set by the software, as in software 1—CL: mean value ± 30% CV; GET: mean value ± 38% CV; SITT: Weibull distribution around the mean value with *α* = 2.92 and *β* = 4.04; or were assigned manually based on a comprehensive literature search as in software 2: lognormal distribution with geometric standard deviation of 1.3 for CL, GET: uniform distribution of 0.2–1.9 h in adults (49,50) and 0.2–2.1 h in children (51–54), and SITT: normal distribution with a mean value of 4 ± 1 h in both (49,55).

### Clearance Scaling

The modeling tools used here employ a physiology-based scaling of adult clearance to children. In short, data sets of experimentally obtained clearances of various substances, for which elimination were primarily due to one process, were previously collected and used to develop and validate ontogeny patterns accounting for the maturation of various elimination pathways over the pediatric age, including renal elimination (22,23). These ontogeny profiles are incorporated in the used modeling tools, and employed along age-specific differences in bodyweight, eliminating organs weight and blood flow, and protein binding in order to scale adult clearance value (model input) to children of different age. This physiology-based scaling of clearance was shown to accurately predict clearance in children from birth to adolescence (22,23) and was found superior to that of allometric scaling.

### Evaluation of Model Performance

Visual predictive checks for superimposed predicted and observed plasma concentration-time profiles, and goodness of fit plots were used for the graphical analysis of model results. Moreover, the area under the plasma concentration-time curve from the first to the last concentration point (AUC_last_), the maximal concentration (*C*_max_) with the time to reach it (*t*_max_), and the elimination-rate constant (*k*_e_) were calculated *via* a noncompartmental analysis for each observed profile and its corresponding predicted value from each model. AUC_last_ was calculated *via* the trapezoidal method, *k*_e_ as the slope of the last three concentrations on a natural logarithmic scale (in children: only for plasma profiles measured over at least 10 h, *n* = 66/80), whereas *C*_max_ and *t*_max_ were manually determined as in definition. An observed/predicted ratio (ratio_Obs/Pred_) was then calculated and the final results were reported as mean ratios_(Obs/Pred)_ with a nonparametric 95% confidence interval (CI) derived from 10,000 bootstrap repetitions. A twofold error range from the observed values for model predictions was set as a reference. Such a range is commonly reported by other researchers and is considered appropriate for a predictive model (14,16,19). Finally, percentage error (PE) and absolute percentage error (APE) were calculated for every concentration point in each drug administration in adults and children as follows:

1 $$ \mathrm{PE}=\frac{\left({C}_{\mathrm{PRED}}-{C}_{\mathrm{OBS}}\right)}{C_{\mathrm{OBS}}}\times 100\% $$

2 $$ \mathrm{APE}=\frac{\left|{C}_{\mathrm{PRED}}-{C}_{\mathrm{OBS}}\right|}{C_{\mathrm{OBS}}}\times 100\% $$

To numerically describe the model accuracy and precision, the median percentage error (MDPE) and median absolute percentage error (MDAPE; with a nonparametric 95% CI derived from 10,000 bootstrap repetitions) were reported, respectively, as suggested in Sheiner and Beal (56). All of the previously mentioned calculations were done using MATLAB 2012a (57).

## RESULTS

### Simulation Results in Adults

**Fig. 3:**
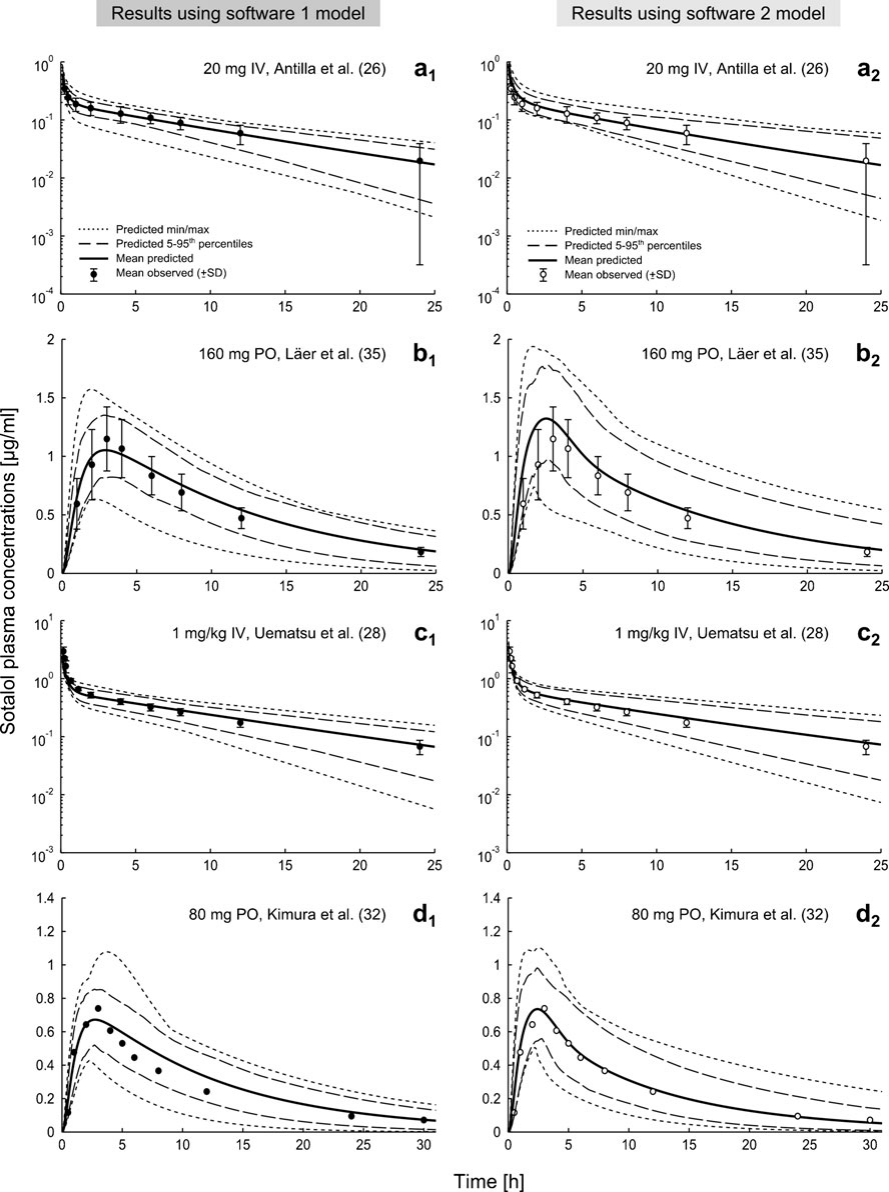
Comparison of predicted (*lines*; mean, 5–95th percentiles, min/max) and mean observed (*dots*; ±SD) concentrations of IV and oral sotalol after various dosing in both Caucasians (**a**, **b**) and Asians (**c**, **d**). Simulations were performed using software 1 (SIMCYP®, *left column*, *filled circles*) and 2 (PK-SIM®, *right column*, *empty circles*). Observed data are obtained from Refs. (26,28,32,35)

**Fig. 4:**
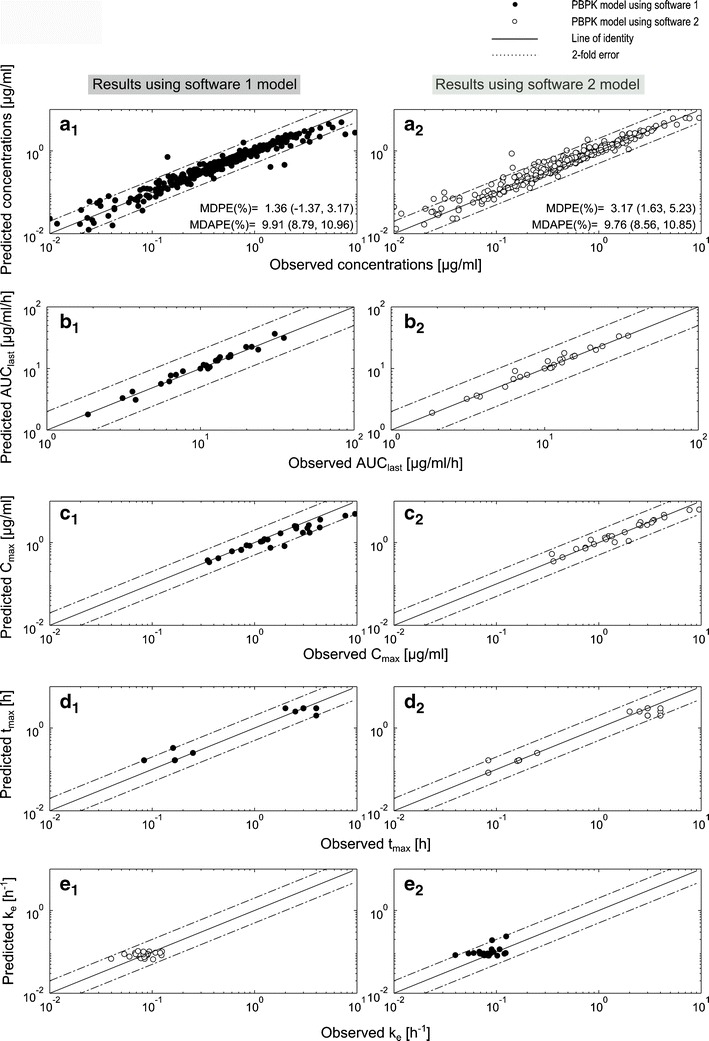
Goodness of fit plots for simulations of adult data by both sotalol PBPK models. **a** Predicted *vs*. observed concentrations plot, **b**–**e** predicted *vs*. observed AUC_last,_ *C*_max_, *t*_max_, and *k*_e_ plots. Results are obtained by using software 1 (SIMCYP®, *left column*, *filled circles*) and software 2 (PK-SIM®, *right column*, *empty circles*). *Line*, line of unity; *dashed lines*, twofold error range; *MDPE* median percentage error (95% CI), *MDAPE* median absolute percentage error (95% CI)

A comparison between mean simulated and mean observed plasma concentration-time profiles for four representative data sets in adults is shown in Fig. 3 (26,28,32,35). In general, the two presented models were able to accurately describe sotalol exposure after IV and oral application over a total dose range of 20 to 320 mg (0.2–4.5 mg/kg BW) and for both Caucasians and Asians. The resulted AUC_last_ ratios_(Obs/Pred)_ were within 0.8–1.25 in 100% (27/27) and in 92.6% (25/27) of the observed values using software 1 and 2, respectively, and with all predictions contained within the range 0.5–2. Moreover, the adult model did not show any difference in the predictability of sotalol exposure after IV or oral application. The mean AUC_last_ ratio_(Obs/Pred)_ for all simulated data sets using software 1 was 0.997 and 0.94 after IV and oral applications, respectively. These results were similar using software 2 as the mean AUC_last_ ratio_(Obs/Pred)_ was 0.94 for the IV and 0.987 for the oral application. Figure 4 shows the predicted *vs*. observed plots for plasma concentrations, AUC_last_, *C*_max_, *t*_max_, and *k*_e_.

The calculated numerical metrics indicate good accuracy by both models. Software 1 showed no bias with a MDPE value (95th bootstrap CI) of 1.36% (−1.37 to 3.17), in comparison to a slight bias of 3.17% (1.63–5.23) in the model generated using software 2, which is, however, minimal and has no clinical relevancy. Finally, both models showed similar precision (deviation less than 10%) as the MDAPE for all predicted concentration points was 9.91% (8.79–10.96) and 9.76% (8.56–10.85) using software 1 and 2, respectively (Fig. 4).

### Simulation Results in Pediatrics

**Fig. 5:**
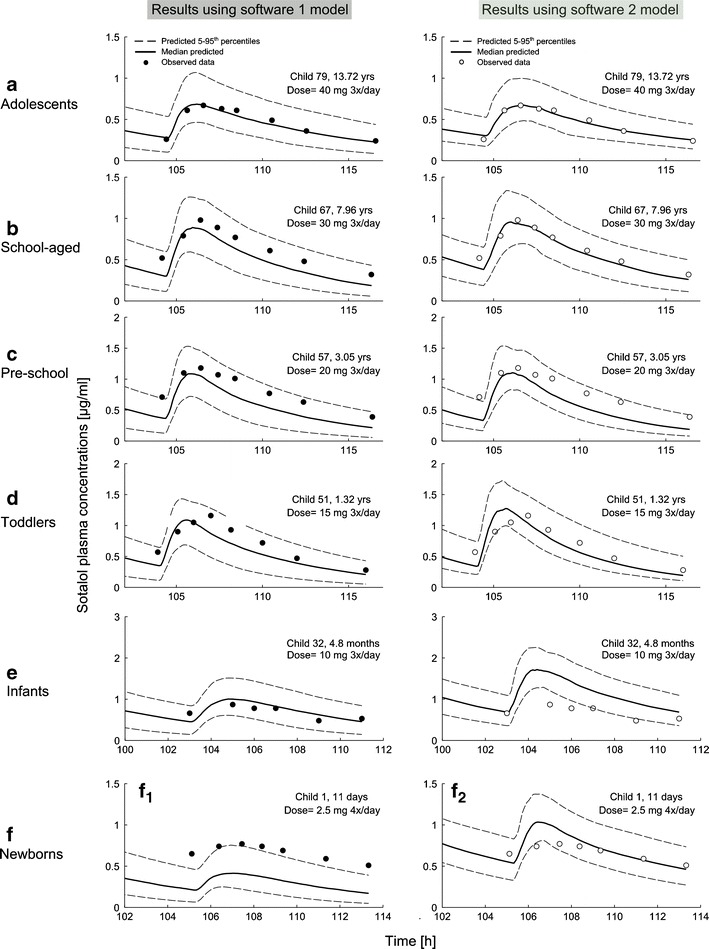
Comparison of predicted (*lines*; median, 5–95th percentiles) *vs*. individual observed (*symbols*) plasma concentrations in six representative pediatric patients from adolescents (**a**) to neonates (**f**), after various dosing of oral sotalol. Predictions were made using software 1 (Simcyp®, *left*, *filled circles*) and 2 (PK-SIM®, *right*, *empty circles*). Observed data are taken from Refs. (37)

Using both modeling software applications, the extrapolated model corresponding to the pediatric population showed acceptable correlated predictions to *in vivo* data in adolescents down to infants, with a pronounced deviation in neonates. A comparison between median simulated and observed plasma profiles for six representative pediatric patients of each age group is shown in Fig. 5 (37).

**Fig. 6:**
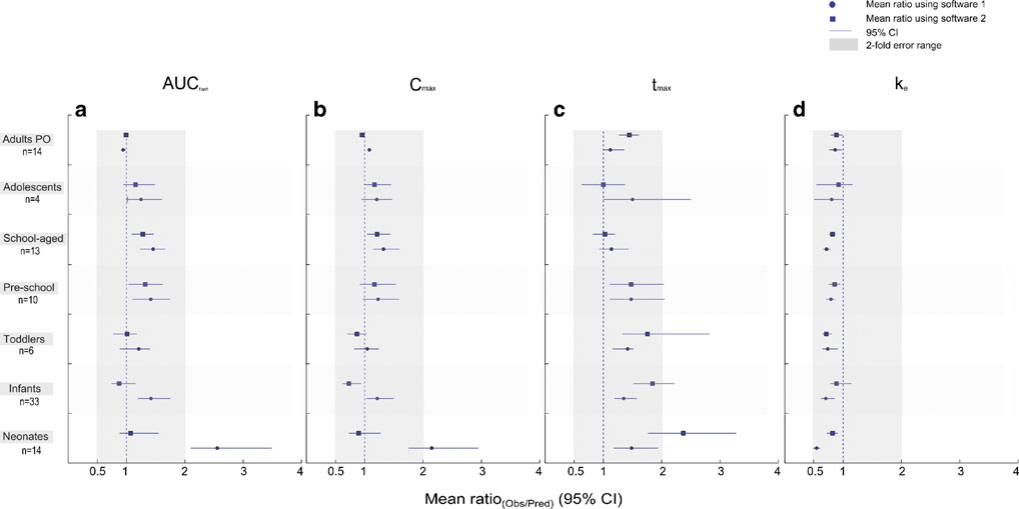
Comparison between the observed and predicted values of **a** the area under the plasma concentration-time curve (*AUC*_*last*_), **b** maximum concentration (*C*_*max*_), **c** time of the maximum concentration (*t*_*max*_), and **d** the elimination-rate constant (*k*_*e*_) in adults oral studies and in children. Results are presented as mean ratios in each age group (*symbols*—*circles* for software 1 results, *squares* for software 2 results) with a 95% confidence interval (*horizontal lines*)

Only in neonates were the both models unable to predict a mean ratio_(Obs/Pred)_ of all four PK parameters within the predefined twofold error range (Fig. 6). Using software 1, the mean ratios_(Obs/Pred)_ were 2.56 (95% CI, 2.10–3.49) and 2.15 (95% CI, 1.77–2.99) for AUC_last_ and *C*_max_, respectively. Using software 2, the mean ratio_(Obs/Pred)_ of *t*_max_ was 2.37 (95% CI, 1.76–3.25). The elimination-rate constant was reasonably predicted by both models as indicated by a mean ratio of 0.55 and 0.81 for the software 1 and the 2 model, respectively.

In all remaining age groups, the mean ratios_(Obs/Pred)_ for the chosen PK parameters were within a twofold error range irrespective of the model used, which indicates a good predictive performance and a proper description of the age-related pharmacokinetic changes of sotalol. The 95% CI of the mean ratios for all PK parameters was contained within the range of 0.5 to 2, except for *t*_max_ in some age groups (Fig. 6).

**Fig. 7:**
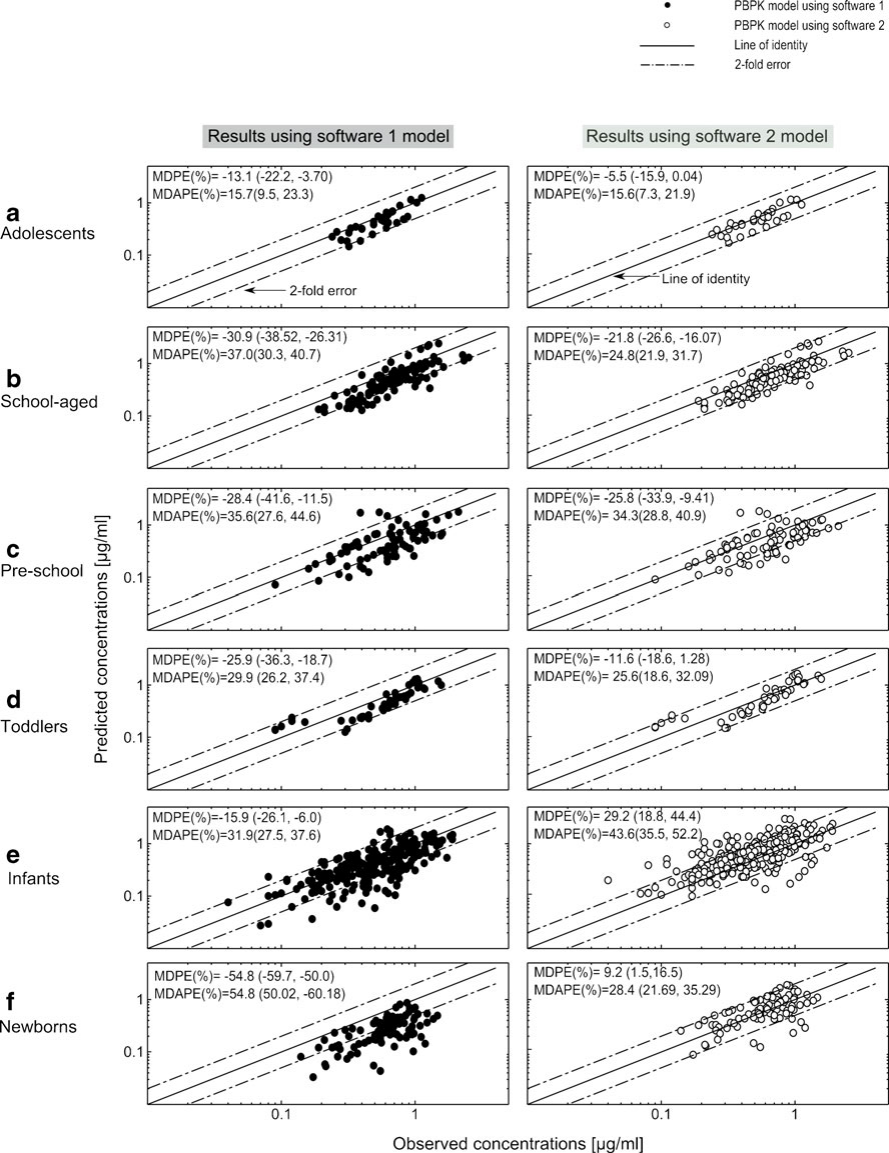
Median predicted *vs*. individual observed concentration plots for 80 pediatric patients stratified in 6 pediatric age groups from **a** adolescents to **f** neonates. Results are obtained by using software 1 (SIMCYP®, *left column*, *filled circles*) and 2 (PK-SIM®, *right column*, *empty circles*). *Line*, line of unity; *dashed lines*, twofold error range; *MDPE* median percentage error (95% CI), *MDAPE* median absolute percentage error (95% CI)

Furthermore, both models showed a general tendency to underestimate sotalol concentrations as seen with the negative MDPE values in almost all age groups and the lower accuracy and precision values when compared with the adult model (see Fig. 7). The least accuracy in predicting individual concentration points was seen in neonates for software 1 (MDPE = −54.8%) and in infants for software 2 (MDPE = 29.2%), whereas for both models the highest accuracy was seen in adolescents. On the other hand, the pediatric model imprecision was less than 40% in all groups except in neonates using software 1 (MDAPE = 54.8%) and in infants using software 2 (MDAPE = 43.6%), with the best model precision seen in adolescents using both software (MDAPE = 15.7%, 15.6% for software 1 and 2, respectively).

## DISCUSSION

Whole-body PBPK models for sotalol, an orally given drug, were developed using two specialized modeling software tools. The presented models were able to successfully describe sotalol pharmacokinetics in adults and over a wide range of the pediatric age, except in neonates. The results obtained by both models were comparable and showed differences only in children under 1 year of age.

Following the methodological approach, PBPK models of sotalol were first developed and evaluated in adults. Both models were able to accurately and reliably predict sotalol exposure after a wide range of IV and oral dosing (Figs. 3 and 4), which indicate that they adequately captured the major processes driving sotalol pharmacokinetics. The initial development and validation of the model in adults present a modeling strategy that forms a solid basis for age extrapolation to increase the accuracy of the pediatric model predictions. Such a strategy is common in the development of pediatric models and is already used by other researchers (16,17,19).

In adolescents down to infants, the pediatric models seemed to properly reflect the age-related changes in sotalol pharmacokinetics, as indicated by the adequate description of the experimental plasma profiles, which was further supported by the numerical metrics and a good prediction of AUC_tlast_, *C*_max_, *t*_max_, and *k*_e_ indicated by a mean ratio_(Obs/Pred)_ within a twofold error range (Figs. 5, 6, and 7). It was only for t_max_, where the calculated 95% CI exceeded the twofold error range in some age groups; however, this could be explained, except for infants, by the relatively low number of included children. Comparing our results with the other available pediatric model for the oral application, Parrot *et al*. reported in a model for the oral oseltamivir and its metabolite a predicted AUC in infants that was within a twofold range of the observed value (19), which resembles our finding using both software for the same age group.

In neonates, no model was able to predict a mean ratio_(Obs/Pred)_ for all reported PK parameters (AUC_last_, *C*_max_, *t*_max_, and *k*_e_) within the predefined twofold error range, and, therefore, the results were judged as inadequate (Fig. 6). The noticed deviation was seen for parameters reflecting the extent and rate of drug absorption (AUC_last_, *C*_max_, or *t*_max_) rather than drug disposition (*k*_e_). In the previously mentioned model by Parrot *et al*., a difference of more than twofold upon AUC prediction of oseltamivir and its metabolite in neonates was obtained, which is similar to our findings (19). A mean ratio_(Obs/Pred)_ higher than two for any PK parameter—as in our results—implies that the model predicts a value that is, on average, less than half of the experimentally observed one. Nevertheless, the clinical relevancy of such results should be eventually judged taking additionally into consideration the intended use of the generated data, and the allowable error by the drug (*e.g*., low for drugs with narrow therapeutic window). For example, this deviation seen in neonates would be of more clinical relevancy if the model is intended to be used to make dose recommendations than to suggest sampling times. In the former case, any recommendations in neonates based on such low predicted AUC, in comparison to the observed, could lead to recommending higher therapeutic doses than necessary with potential toxicity (*e.g*., torsades de pointes with sotalol) as a clinical consequence.

Both of the presented models performed similarly in adults and in almost all pediatric age groups with the only discrepancy seen in children less than 1 year of age. First, whereas the software 1 model tended to under-predict plasma concentrations in all age groups, including infants and neonates (negative MDPE), the software 2 model under-predicted plasma concentrations only in children over 1 year of age. Finally, in neonates, the software 1 model was unable to accurately predict the extent of drug absorption as indicated by a mean ratio_(Obs/Pred)_ exceeding 2 for AUC_last_ and *C*_max_, whereas the software 2 model did not adequately predict the rate of drug absorption (*t*_max_) in the same age group.

We suggest that the inaccuracy of the predictions seen in neonates is attributed to the absorption rather than elimination or distribution process. This is because the major factors that influence sotalol disposition (*e.g*., maturation of the renal function, the age-related differences in body composition, tissue volumes, and blood flows) are well characterized over the entire pediatric age range and are successfully implemented in the used modeling tools, which should make the scaled information (*e.g*., clearance) a good estimate. In this exercise, both models were able to acceptably predict the elimination-rate constant in all pediatric age groups (Fig. 6). However, the marginally accepted low mean ratio_(Obs/Pred)_ for *k*_e_ in neonates, using software 1, indicate relatively high predicted values that are most probably attributed to high predicted clearance in this age group. As a result, the potential influence of any inaccuracy in clearance scaling on the obtained results in neonates should not be completely excluded.

By contrast, the absorption process is more complex and involves many factors apart from the pharmaceutical formulation of the drug. The first set of them are anatomical and physiological such as gastrointestinal organs volume and blood flow, radius, length and effective surface area, pH, GET, SITT, metabolizing enzymes, transporters, and fluid secretion. Influenced, in part, by difficulties in obtaining age-specific information in the literature, an age-specific value is not incorporated for all of these factors in the pediatric absorption models integrated within the used modeling tools, which makes them an aspect to improve (Table III). For instance, the ADAM model used by software 1 to predict the oral drug absorption is under further improvement to fill such gaps with age-specific values, *e.g*., for metabolizing enzymes, transporter, pH profile, and volume of secreted fluids. As sotalol is not metabolized, not actively transported throughout the gastrointestinal tract, and highly soluble, an age-related change in the pH profile could be the sole possible factor from this list to influence its extent of absorption.

**Table III:** List of the Anatomical, Physiological, and Drug-Specific Parameters that Are Involved in the Drug Absorption with the Availability of Corresponding Age-Specific Values, as Default, in the Used Pediatric Absorption Models

	Software 1	Software 2
Anatomical and physiological parameters		
GIT organs volumes	Scaled with age-specific data	Scaled with age-specific data
GIT organs blood flows	Scaled with age-specific data	Scaled with age-specific data
Radius of the intestinal segments	Scaled with age-specific data	Scaled with age-specific data
Length of the intestinal segments	Scaled with age-specific data	Scaled with age-specific data
Effective surface area of intestinal segments	Scaled with age-specific data	Scaled with age-specific data
Gastric pH	Not scaled (adult values)^a, b^	Not scaled (adult values)^a^
Intestinal pH	Not scaled (adult values)^a^	Not scaled (adult values)^a^
Gastric emptying time	Not scaled (adult values)^a^	Not scaled (adult values)^a^
Small intestinal transit time	Not scaled (adult values)^a^	Not scaled (adult values)^a^
Intestinal enzyme ontogeny	Scaled for CYP3A4 and UGT	Scaled for CYP3A4 and UGT
Intestinal enzyme abundance	Not scaled (adult values)^c^	Not scaled (adult values)^c^
Intestinal transporter ontogeny	Not scaled (adult values)^c^	Not scaled (adult values)^c^
Intestinal transporter abundance	Not scaled (adult values)^c^	Not scaled (adult values)^c^
Fluid secretion volume	Not scaled yet^b^	Volumes are scaled according to length and radius of intestinal segments
Drug-specific parameters		
Molecular weight of the molecule	Unchanged	Unchanged
p*K*_a_ value	Unchanged	Unchanged
Lipophilicity	Unchanged	Unchanged
Solubility	Unchanged	Unchanged
Permeability coefficient in the gut wall	Unchanged	Unchanged

This list does not include factors related to the pharmaceutical formulation

^*a*^Values and variability ranges could be manually assigned. In software 2, various distribution types of the variability could be assigned during the simulations of virtual populations

^*b*^To be incorporated in the upcoming versions

^c^No sufficient literature of specific pediatric data

The second set of parameters is drug dependent such as solubility, p*K*_a_, volume/size of the molecule and its intestinal permeability. Concerning the latter, Yang *et al*. suggested paracellular transport to play a major role in sotalol permeability (47). Dahan *et al*. stated that, in adults, sotalol shows a unique permeability pattern as a combination of a basic moiety, with p*K*_a_ and logP values within a critical range, and that sotalol permeability in the distal small intestine is high and compensates for its low permeability in the proximal segments (58), which explains the low *in vitro* measured jejunal permeability of sotalol, the BCS class I drug. In recognition of this information, the predictions of software 1 model in neonates would have been improved, if a higher permeability of sotalol is assumed and incorporated into the model, as it has already been reported that paracellular transport is higher in neonates than in adults because of wider tight junctions (59). Conversely, the findings of Dahan *et al*. highlight the role of higher pH values in sotalol absorption, and points out a rationale to incorporate different values of permeability throughout the intestinal segments, which could improve software 2 model predictions. Running simulation scenarios by the presented PBPK pediatric models would clarify, reject, or confirm these assumptions and would help in detecting the most influential factor on sotalol absorption.

### Limitations

The two presented models were not completely identical in their input parameters (Table I); however, the different input values of B/P ratio and intestinal permeability measures resulted eventually in similar *V*_ss_ and bioavailability values and, thus, are not likely to be responsible for any major finding. Second, although the work presented here would contribute to a better understanding, and thus to a more correct use of the PBPK model-generated data in the pediatric population, sotalol is a drug with a relatively simple pharmacokinetic profile, *i.e*., renally excreted, not metabolized, unbound to plasma proteins, and is not known to be actively transported (24,25); therefore, the used software tools may not necessarily do as well with predictions for drugs with more complex pharmacokinetics. In addition to that, sotalol is a BCS class I drug with a high solubility and a high permeability profile, which means that this exercise will give no information on the accuracy of the drug absorption in the lower intestinal segments and its age dependence. As a result, further examples with drugs that possess different pharmacokinetic profiles (*e.g*., hepatic elimination with first-pass effect or with involvement of transporters) and physicochemical properties (*e.g*., of different BCS grouping) are still needed.

### Implications and Generalizations

This work was not designed to investigate new insights on the pharmacokinetics of sotalol, as they are already extensively studied in both adults and children (24–37). However, the current work is planned to support a future pediatric clinical trial, which will aim to develop a safe IV dosing regimen as a substitution of oral sotalol in children with supraventricular tachycardia by providing the necessary sampling times for an optimal pharmacokinetic analysis. Additionally, such validated models could play a role in supporting clinical decision making in individual patients, for example, with reduced renal function.

The obtained results in infants through adolescents indicate a good model predictability and, thus, substantiate the use of PBPK models to generate data *a priori* for this age group, saving time, effort, and resources. This could be probably generalized to other orally given drugs that share a similar pharmacokinetic behavior as sotalol. Alternatively, the lower model predictability of sotalol pharmacokinetics seen in neonates indicates the need for a more cautious use of model-generated data in this age group, acknowledging that the final judgment depends on the purpose of the model and the properties of the modeled drug.

## CONCLUSIONS

In summary, the PBPK models presented in this article have shown good predictability of observed data in adults and in almost all pediatric age groups, except in neonates where a lower predictive performance was seen, which indicates a more cautious use of model-generated data in this age group. These results encourage the use of PBPK models, especially when adult data are available, to predict oral drug exposure in a wide range of pediatric age, which can aid in supporting pediatric clinical trials, and, potentially, the clinical decision making for individual children.
